# Deeper Trophoblastic Invasion and More-Promotive Vascular Remodeling in Tubal Isthmic Pregnancy Compared with Ampullary Pregnancy: A Retrospective Clinicopathological Study

**DOI:** 10.1007/s43032-025-01841-7

**Published:** 2025-03-17

**Authors:** Li Yan, Yang Wang, Huiyu Zhang, Bangchun Lu, Jinglan Liu, Yamei Li, Juan Li, Jian Zhang

**Affiliations:** 1 Present Address: Department of Obstetrics and Gynecology, International Peace Maternity and Child Health Hospital, School of Medicine, Shanghai Jiaotong University, 910 Hengshan Road, Shanghai, Xuhui District China; 2 Shanghai Key Laboratory Embryo Original Diseases, Shanghai, China; 3 Present Address: Department of Pathology, International Peace Maternity and Child Health Hospital, School of Medicine, Shanghai Jiaotong University, 910 Hengshan Road, Shanghai, Xuhui District China

**Keywords:** Tubal pregnancy, Tubal isthmic pregnancy, Tubal ampullary pregnancy, Chronic inflammation of the tube, Salpingitis isthmica nodosa

## Abstract

To explore clinical and pathological characteristics of tubal isthmic pregnancy. A single-center, retrospective clinicopathological cohort study was conducted in women diagnosed with tubal pregnancy that underwent salpingectomy in International Peace Maternal and Child Health Care Hospital from January 2018 to April 2023. A total of 598 eligible women with tubal pregnancy were included in the analysis, including 75 women with isthmic pregnancy and 523 women with ampullary pregnancy. Among women with isthmic pregnancy, 29 (38.67%) had trophoblastic infiltration into the muscular layer, while 46 (61.33%) had infiltration into the serosal layer. Meanwhile 66 (12.62%) women, 258 (49.33%) women, and 199 (38.05%) women had trophoblastic infiltration into the mucosal layer, muscular layer, and serosal layer of the ampullary pregnancy, respectively. The study found that women with isthmic pregnancy had enhanced trophoblastic activity, deeper vascular remodeling, increased trophoblastic infiltration into the deep wall of the tube, higher serum beta subunit of human chorionic gonadotropin (β-hCG) levels, and a higher probability of clinical manifestations. Additionally compared with ampullary pregnancy, the occurrence of isthmic pregnancy was positively correlated with salpingitis isthmica nodosa (AOR = 3.62, 95% CI 1.45–9.00, *P* < 0.01), and negatively correlated with the presence of chronic tubal inflammation (AOR = 0.24, 95% CI 0.09–0.64, *P* < 0.01). Salpingitis isthmica nodosa, not chronic inflammation of the fallopian tubes, may be a risk factor for isthmic pregnancy. Compared to ampullary pregnancy, isthmic pregnancy exhibits a more profound trophoblastic vascular remodeling of the fallopian tube and a greater tendency to infiltrate the deep layers of the tubal wall. These characteristics render it more concealed and perilous, underscoring the importance of early recognition and diagnosis.

## Introduction

Tubal pregnancy, a common form of ectopic pregnancy (EP), encompasses various subtypes, with ampullary pregnancy being the most prevalent, accounting for approximately 75% of cases, followed by isthmic, interstitial, and fimbrial pregnancies, which are comparatively rarer [1–3]. The rupture of an ectopic pregnancy constitutes a significant contributor to maternal mortality in early pregnancies, responsible for approximately three-quarters of such tragic cases [1, 4, 5]. Consequently, the early detection, diagnosis, and comprehensive investigation into its etiology and pathogenesis have long been pivotal in this field of study. The site of EP implantation holds particular significance, as it can influence the severity of the condition [2, 6]. Literature has consistently highlighted that isthmic pregnancy carries a considerably higher risk of rupture compared to ampullary pregnancy, primarily attributed to the narrow and non-expandable characteristics of the isthmic segment [2, 7, 8]. Additionally, previous study has noted the frequent destruction of the mucosal layer in isthmic pregnancy [6], prompting further inquiry into whether this histopathological pattern renders ectopic pregnancy more susceptible to rupture. Nevertheless, the research on isthmic pregnancy remains limited, and its clinical and pathological features have yet to be fully elucidated.

The multifaceted nature of the etiology of tubal pregnancy is widely recognized [9–11]. Pelvic inflammatory disease (PID), comprising salpingitis and salpingo-oophoritis, stands out as the primary risk factor for tubal pregnancy [12–15]. Studies have unveiled that chronic inflammation within the fallopian tube leads to an upregulation of inflammatory factors, a downregulation of anti-inflammatory factors, and impaired smooth muscle contraction, thereby retarding embryo transport and promoting implantation within the tube [16–19]. Notably, extant literature on the risk factors associated with ectopic pregnancy underscores a significant correlation with the presence of salpingitis isthmica nodosa (SIN) and isthmic pregnancy [20, 21]. Diverging from chronic inflammation, SIN represents a non-inflammatory pathological state wherein the fallopian tube epithelium extends into the myometrium, forming cystic dilated diverticula and resulting in nodular thickening of the fallopian tube [20]. This condition not only compromises its peristaltic function, but also contributes to infertility, tubal pregnancy, and recurrent tubal pregnancy [21]. However, previous pathological studies on this subject are limited in scope and sample size [22–25], leaving the question of whether different segments of the fallopian tube pregnancies share similar risk factors still unanswered. Furthermore, the potential relationship between chronic inflammation of the fallopian tube and the implantation site of tubal pregnancy remains unexplored.

In light of these gaps in knowledge, we conducted a retrospective clinicopathological study to compare isthmic pregnancy with tubal ampullary pregnancy, with the aim of elucidating the clinicopathological characteristics of isthmic pregnancy.

## Materials And Methods

### Ethical Approval and Data Collection

This study received approval from the Ethics Review Committee of the International Peace Maternal and Child Health Care Hospital (IPMCH) affiliated with Shanghai Jiaotong University School of Medicine, China (Approval No: GKLW 2019–69). All data were collected anonymously, and informed consent was waived by the Ethics Review Committee due to the retrospective design.

### Study Population

Our study population consisted of women diagnosed with ampullary or isthmic tubal pregnancy who underwent salpingectomy at the IPMCH between January 2018 and April 2023. Participants were identified through a systematic review of medical records and histopathological reports within our institution's database. To ensure eligibility, we employed the following criteria:

Inclusion Criteria:

1. Women with a confirmed diagnosis of ampullary or isthmic tubal pregnancy.
2. Women who had undergone a salpingectomy procedure at IPMCH.

Exclusion Criteria:

1. Cases with incomplete histopathological data, such as those where the implantation site or trophoblastic infiltration could not be determined.
2. Ectopic pregnancies occurring in other locations, such as the cornual region, the interstitial segment, or the fimbrial end of the fallopian tube.
3. Women with borderline or malignant reproductive system tumors.

#### Protocol of Management of Ectopic Pregnancies

At our institution, the management of ectopic pregnancies is guided by the Chinese Expert Consensus on the Diagnosis and Treatment of Tubal Ectopic Pregnancy [26]. The management of ectopic pregnancy in the consensus primarily includes the following approaches, tailored to the patient’s clinical presentation, hemodynamic stability, and desire for future fertility:

##### Expectant Management

This is considered for selected cases with minimal symptoms, declining beta subunit of human chorionic gonadotropin (β-hCG) levels, and no evidence of rupture. Close monitoring of β-hCG levels and ultrasound findings is required to ensure resolution.

##### Medical Management

Methotrexate (MTX) therapy is recommended as a first-line treatment for stable patients with unruptured ectopic pregnancy, meeting specific criteria (e.g., β-hCG < 5000 mIU/mL, ectopic mass size < 3–4 cm, no fetal cardiac activity). MTX can be administered as a single-dose or multi-dose protocol depending on the patient’s condition and response.

##### Surgical Management

Surgery is indicated for hemodynamically unstable patients, cases of ruptured ectopic pregnancy, or those not suitable for or failing medical management. Laparoscopic surgery is preferred over laparotomy whenever feasible, with options including salpingectomy (removal of the affected tube) or salpingostomy (preserving the tube) based on the patient’s reproductive preferences and the extent of damage.

##### Post-treatment Follow-up

Regular monitoring of β-hCG levels is essential to confirm the resolution of the ectopic pregnancy. Counseling on future pregnancy planning and risk factors for recurrent ectopic pregnancy is also emphasized.

## Data Collection

### Collection of Clinical History

We gathered baseline characteristics from the clinical database, encompassing age, menstrual cycle, last menstrual period (LMP), pregnancy history (gravidity, parity, miscarriage or abortion, previous ectopic pregnancy), and relevant clinical information about the current pregnancy, including clinical symptoms, mode of conception, and contraception methods.

### Clinical Parameters

Preoperative serum β-hCG levels, final preoperative ultrasound findings (including the presence of the ectopic embryo sac, germ or fetal cardiac activity), and surgical findings (tubal rupture, active bleeding of the fallopian tube, pelvic adhesions, etc.) were also recorded.

### Clinical Examination

Serum β-hCG and transvaginal Doppler ultrasonography were performed by skilled medical professionals following standardized procedures. These examinations provided measurement values for serum β-hCG (IU/L) and key ultrasonic indicators (gestational sac, embryo, fetal heart, and size of adnexal mass). Serum β-hCG was quantified using the Immulite 2000 XPi system, a two-site chemiluminescence immunoassay. Transvaginal color Doppler ultrasound data from Philips IU22, GE Volution E8, and GE Volution E10 were unified by professional doctors. The main ultrasonic manifestations of ectopic pregnancy include an extrauterine gestational sac with a yolk sac and/or a fetal pole that may or may not have fetal cardiac activity[27]. The size of the non-cystic adnexal mass (mm) was determined by measuring its longer axis by transvaginal ultrasound[28].

### Pathological Patterns

Two experienced pathologists, blinded to clinical data, performed histological evaluations. Hematoxylin–eosin-stained sections were obtained from the IPMCH pathology department. Dual immunofluorescence staining was performed to evaluate the stage of vascular remodeling of fallopian tube following the manufacture’s protocols. Briefly, 6 μm sections from the formalin-fixed, paraffin-embedded samples were deparaffinized, rehydrated and processed for antigen retrieval in antigen retrieval solution (pH 8). After inactivation of endogenous peroxidase activity and blocking at room temperature, it was incubated with primary antibody at 4 °C overnight, followed by PBS washing and secondary antibody incubation. Nuclei were stained with DAPI. Primary antibodies used for IF include: HLA-G (79769 T, rabbit monoclonal antibody, 1:100) and SMA (AF20157, mouse monoclonal antibody,1:100). Pathological diagnostic criteria were in accordance with previously published literature [4, 29].

## Histopathological Features Observed

**Fig. 1:**
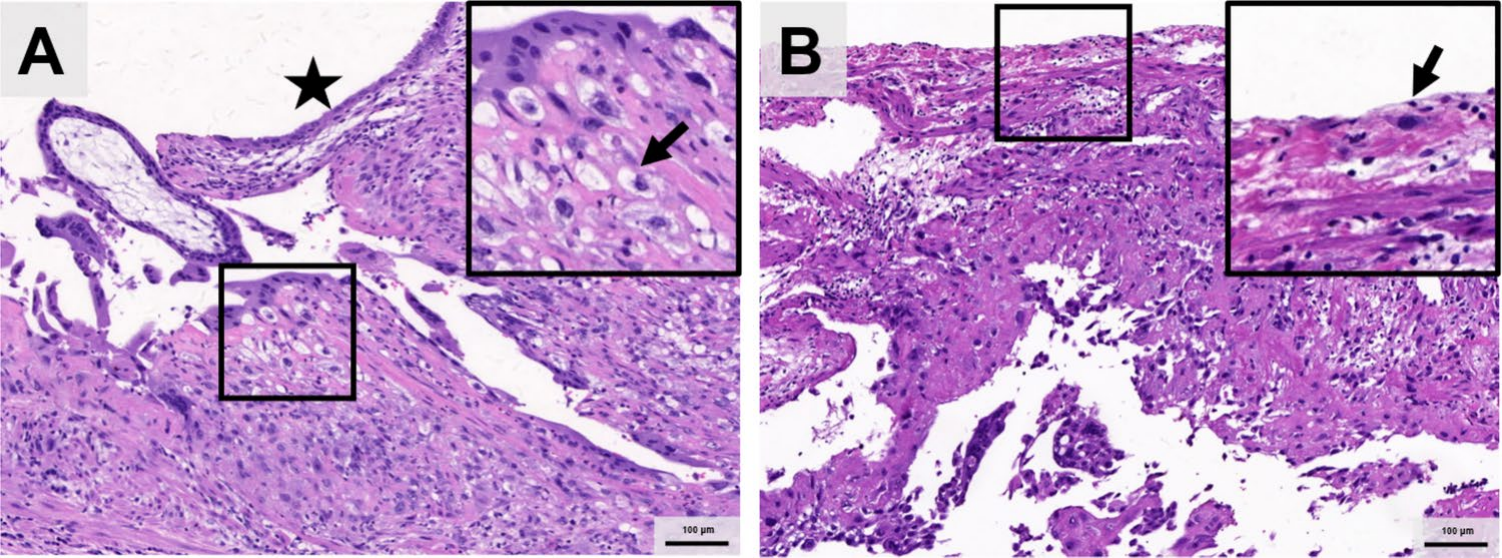
Different infiltration depth of the tubal isthmic pregnancy. **A** Extravillous trophoblast (EVT) infiltrates submucosal muscular layer. The arrow shows the EVT, and the star shows the lumen. **B** Extravillous trophoblast (EVT) infiltrates tubal serosa. The arrow shows the EVT under the serosa epithelium

**Fig. 2:**
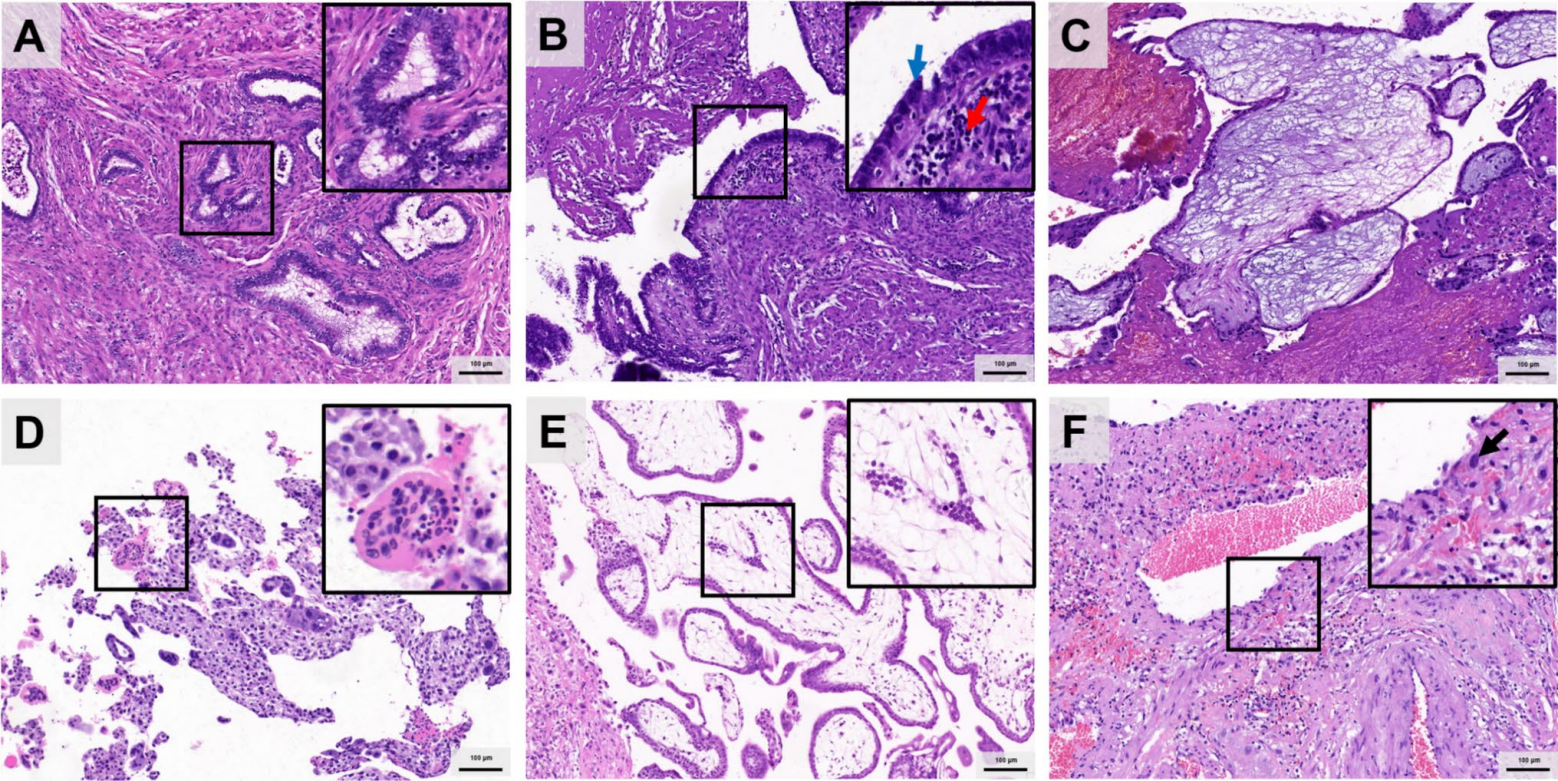
Histopathologic analysis of tubal isthmic pregnancies. **A** Salpingitis isthmica nodosa (SIN). **B** Chronic inflammation of isthmic tubal mucosa. The red arrow indicates the lymphocytes, and blue arrow indicates epithelium. **C** Villous edema, characterized by the interstitial accumulation of liquid and then edema to form different sizes of vesicles. **D** Degenerative syncytiotrophoblast with eosinophilic cytoplasm and condensed heavily stained nuclei. **E** The presence of fetal nucleated red blood cells in villous blood vessels. **F** Spiral artery remodeling, characterized with endovascular trophoblasts move and infiltrate arterial wall, replace the endothelium, and disrupt the muscular lining. The arrow indicates the EVT

The primary histopathological features included:

- Extravillous trophoblastic (EVT) infiltration depth (muscular, serosal layers). (Fig. 1)
- Salpingitis isthmic nodular (SIN). (Fig. 2.A)
- Chronic inflammation of fallopian tubes. (Fig. 2.B)
- Morphology and development of villi (villi edema, trophoblast cell degeneration, presence of fetal nucleated red blood cells in chorionic blood vessels, and vascular remodeling). (Fig. 2.C-F)

**Fig. 3:**
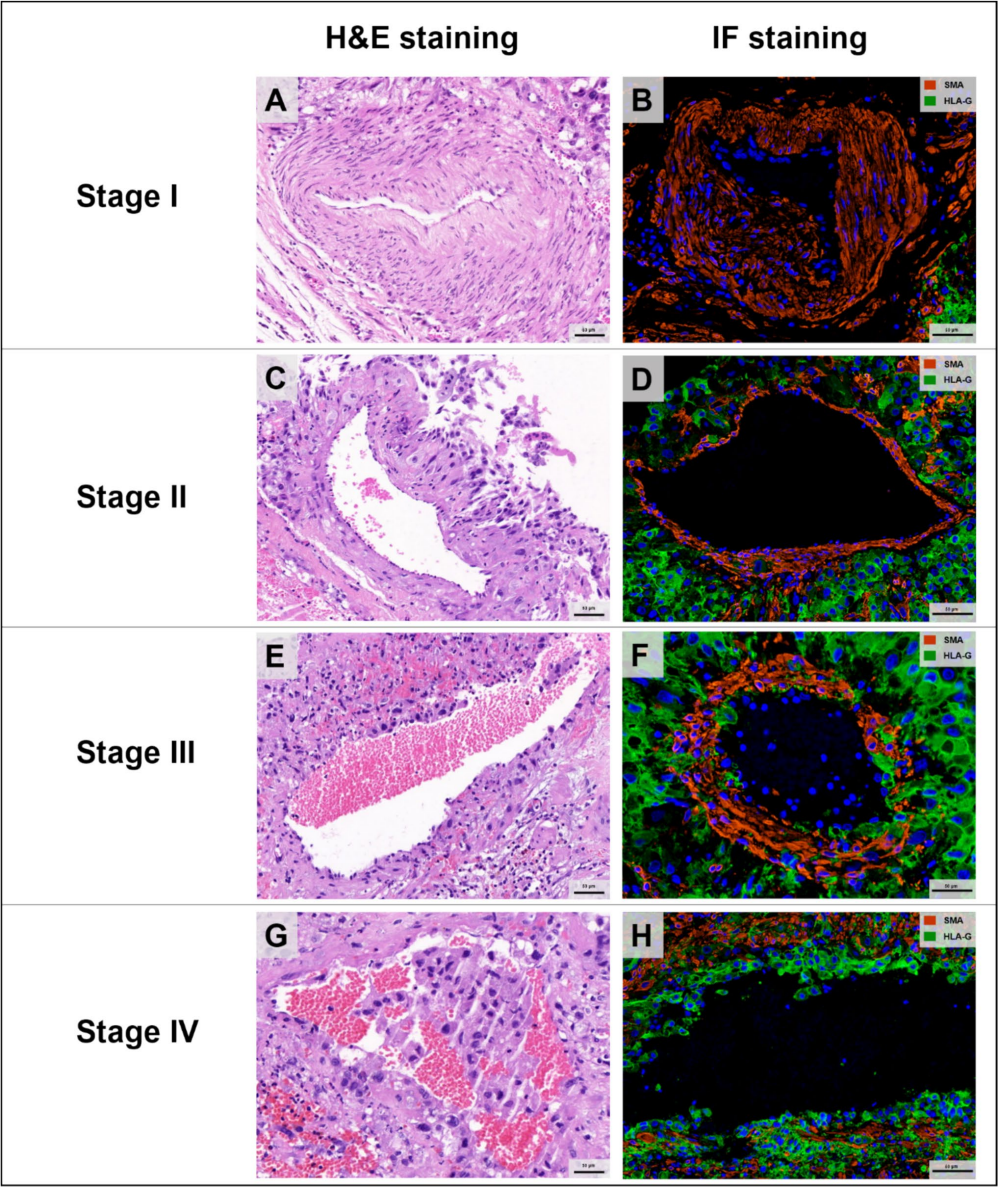
Four stages of vascular remodeling in tubal isthmic pregnancy. **A-B** Stage I. Vessel displayed intact vascular smooth muscle cells (VSMC) layers and endothelium, with no detectable EVTs present. **C-D** Stage II. EVTs infiltrate spiral artery with disruption, disorganization, and partial loss of VSMC. **E–F** Stage III. Vessel exhibited substantial loss of VSMC and endothelium, with EVTs present in the lumen and adherent to the vascular wall. **G-H** Stage IV. Vessel exhibited complete loss of VSMC and endothelium. (H&E staining: Hematoxylin & eosin staining; IF staining: Immunofluorescence staining; SMA (smooth muscle actin) is a marker used to label vascular smooth muscle cells; HLA-G is a marker used to identify EVT cells.)

Vascular remodeling was categorized into four stages based on the degree of endothelial cell and vascular smooth muscle cell (VSMC) disruption [29] (Fig. 3):

- Stage I: Intact intima and media with no detectable EVTs. (Fig. 3.A-B)
- Stage II: VSMC layer disruption with interstitial EVTs accumulation. (Fig. 3.C-D)
- Stage III: Extensive VSMC and endothelial cell loss, with endovascular EVTs adhering to the vessel wall. (Fig. 3.E–F)
- Stage IV: Complete replacement of vessels by extravillous trophoblasts. (Fig. 3.G-H)

## Statistical Analysis

Statistical analyses were conducted using SPSS Version 25.0 (IBM, Armonk, NY, USA). Normality was assessed with the Shapiro–Wilk test. Continuous variables were described as percentages and compared using the chi-square test or Fisher exact probability method. Normally distributed quantitative data were presented as mean [standard deviation] and compared using the student t-test. Non-normally distributed quantitative or graded data were described using median and quartiles and compared using the Mann–Whitney rank test. One-way analysis of variance was performed among the three groups, with homogeneity of variance assessed by the Bartlett test, and clinical independence of groups confirmed. Logistic multivariate regression analysis was conducted to explore potential factors associated with implantation in the ampulla or isthmus, with adjusted odds ratios (AOR) and 95% confidence intervals (95% CI) used to represent statistical differences. Two-tailed p-values < 0.05 were considered statistically significant.

## Results

### Study Participants

**Fig. 4:**
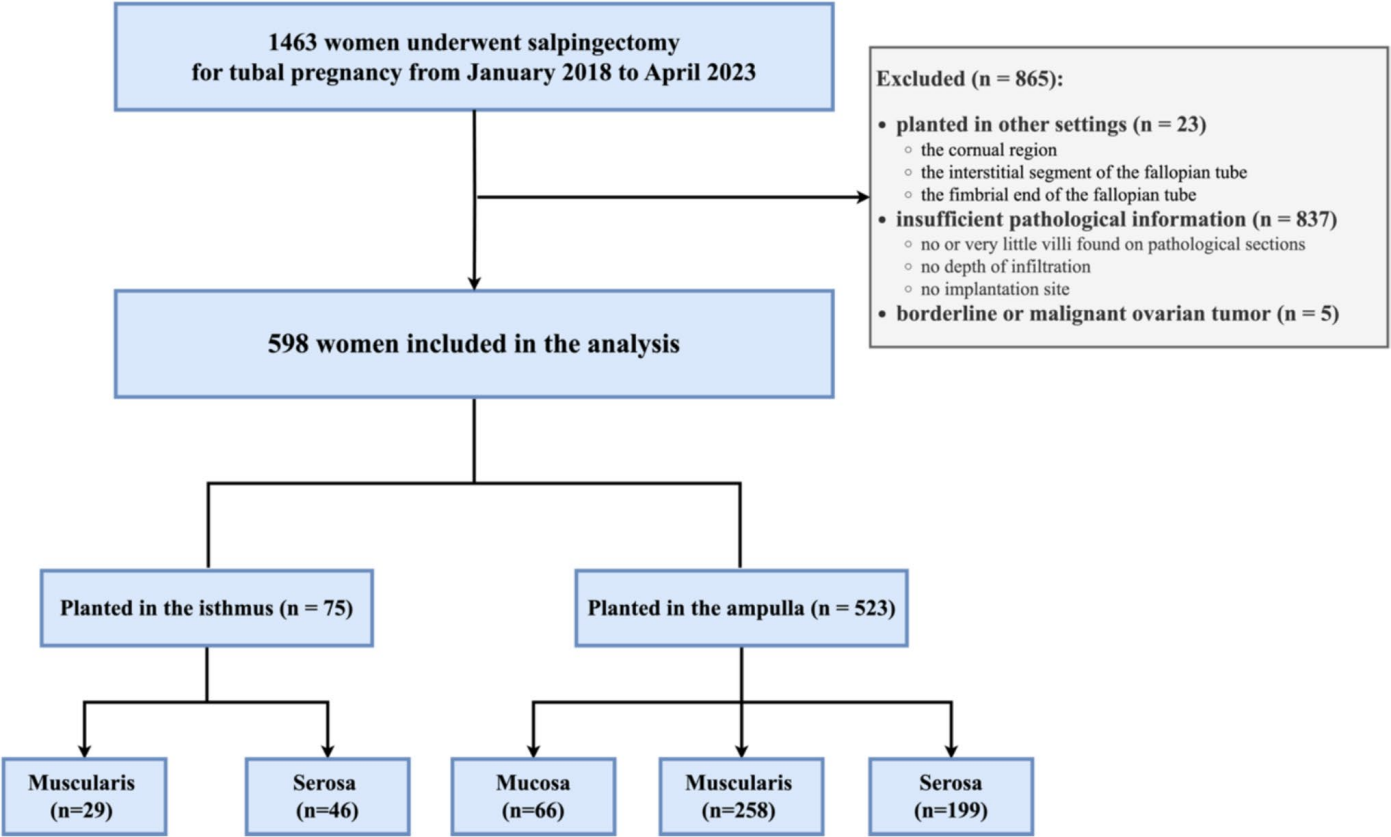
Flow chart

Figure 4 presents a flowchart of our study, outlining the inclusion and exclusion criteria for participants in this research. It also delineates the distribution of participants with isthmic and ampullary pregnancies according to the depth of trophoblast invasion. Specifically, a total of 1463 women underwent salpingectomy due to tubal pregnancy. After applying exclusion criteria, 865 women were excluded, leaving a final cohort of 598 women for analysis. Among them, 75 women were diagnosed with isthmic pregnancy, while 523 women had ampullary pregnancies. Based on the depth of trophoblastic infiltration into the fallopian tube wall, participants were categorized into mucosa, muscular, and serosa groups. In detail, none of the women with isthmic pregnancy and 66 (12.62%) with ampullary pregnancy had trophoblastic infiltration into the mucosa layer, 29 (38.67%) women with isthmic pregnancy and 258 (49.33%) with ampullary pregnancy had trophoblastic infiltration into the muscular layer, while 46 (61.33%) women with isthmic pregnancy and 199 (38.05%) women with ampullary pregnancy had serosal layer infiltration.

### Comparison of Clinical Characteristics

Table 1 presents a comparison of medical history and clinical characteristics. Compared to ampullary pregnancy, isthmic pregnancy exhibited a lower probability of clinical symptoms (80.56% vs. 89.56%, *P* = 0.03), a longer time from last menstrual period (LMP) to surgery (medium 48.00 days vs. 47.00 days, *P* < 0.01), higher β-hCG levels (medium 4722.80 IU/L vs. 4357.00 IU/L, *P* < 0.01), and a lower incidence of tubal surface adhesion on the affected side (9.33% vs. 18.74%, *P* = 0.04). Transvaginal ultrasound also revealed that adnexal mass size was smaller (medium 22.00 mm vs. 25 mm, *P* < 0.01) and germ detection rate was higher (20.00% vs. 11.09%, *P* = 0.04) in isthmic pregnancy. Although not statistically significant, the detection rate of fetal heart rate tended to be higher in isthmic pregnancies compared to ampullary pregnancies (24.00% vs. 15.11%, *P* = 0.06).

**Table 1:** Comparison of clinical characteristics of women between isthmic and ampullary pregnancy

No. (%) / Mean [SD] / M (Q1, Q3)	Isthmic (*n* = 75)	Ampulla (*n* = 523)	*P* value
**Baesline characteristics**			
**Age**			
mean age	32.11 [5.21]	32.65 [4.72]	0.36
**Reproductive history**			
**Gravidity**			
0	18 (24.00)	100 (19.12)	0.54
≥ 1	57 (76.00)	423 (80.88)	
**Parity**			
0	42 (56.00)	248 (47.42)	0.09
≥ 1	33 (44.00)	275 (52.58)	
**Previous spontaneous miscarriage**			
0	28 (37.33)	222 (42.45)	0.72
1	27 (36.00)	169 (32.31)	
≥ 2	20 (26.67)	132 (25.24)	
**Previous tubal pregnancy**			
0	60 (80.00)	413 (78.97)	0.44
1	12 (16.00)	102 (19.50)	
2	3 (4.00)	8 (1.53)	
**Information about this pregnancy**			
**After IVF treatment**			
No	67 (89.33)	496 (94.84)	0.10
Yes	8 (10.67)	27 (5.16)	
**Take LNG-EC during this menstrual cycle**			
No	74 (98.67)	495 (94.65)	0.22
Yes	1 (1.33)	28 (5.35)	
**Have clinical symptoms**			
No	14 (19.44)	54 (10.33)	0.03
Yes	58 (80.56)	463 (88.53)	
**Time from LMP to the onset of clinical symptoms**	35.00 (30.00, 45.00)	36.00 (32.00, 40.00)	0.51^a^
**Time from LMP to surgery**	48.00 (46.00, 62.00)	47.00 (42.00, 53.00)	< 0.01^a^
**Last β-hCG before surgery (IU/L)**	4722.80 (3710.40, 8177.50)	4357.00 (2121.10, 8404.90)	< 0.01^a^
**Failure of methotrexate treatment**	1 (1.33)	12 (2.29)	1.00
**Findings on transvaginal ultrasound**			
**With an intrauterine pregnancy simultaneously**			
No	73 (97.33)	511 (97.71)	1.00
Yes	2 (2.67)	12 (2.29)	
**Size of the adnexal mass on ultrasound (mm)**	22.00 (18.00, 29.00)	25.00 (20.00, 37.00)	< 0.01^a^
**Ectopic fetal heart**			
No	57 (76.00)	444 (84.89)	0.06
Yes	18 (24.00)	79 (15.11)	
**Ectopic embryo sac**			
No	57 (76.00)	431 (82.41)	0.20
Yes	18 (24.00)	92 (17.59)	
**Ectopic germ**			
No	60 (80.00)	465 (88.91)	0.04
Yes	15 (20.00)	58 (11.09)	
**Findings during surgery**			
**Ruptured tube seen during surgery**			
No	66 (88.00)	475 (90.82)	0.53
Yes	9 (12.00)	48 (9.18)	
**Pelvic adhesions**			
No	56 (74.67)	355 (67.88)	0.29
Yes	19 (25.33)	168 (32.12)	
**Adhesion on the surface of the ipsilateral fallopian tube**			
No	68 (90.67)	425 (81.26)	0.04
Yes	7 (9.33)	98 (18.74)	
**Adhesion on the surface of the contralateral fallopian tube**			
No	68 (90.67)	471 (90.06)	1.00
Yes	7 (9.33)	52 (9.94)	
**Active bleeding on the tube seen during surgery**			
No	69 (92.00)	497 (95.03)	0.42
Yes	6 (8.00)	26 (4.97)	

*SD* standard deviation, *M* (Q1, Q3), median (first quartile, third quartile), *LMP* last menstrual period, IVF in vitro fertilization, *LNG-EC* levonorgestrol-emergency contraception, *β-hCG* beta-human chorionic gonadotropin

^a^Tested by Mann–Whitney U Test

### Comparison of Pathological Patterns

**Fig. 5:**
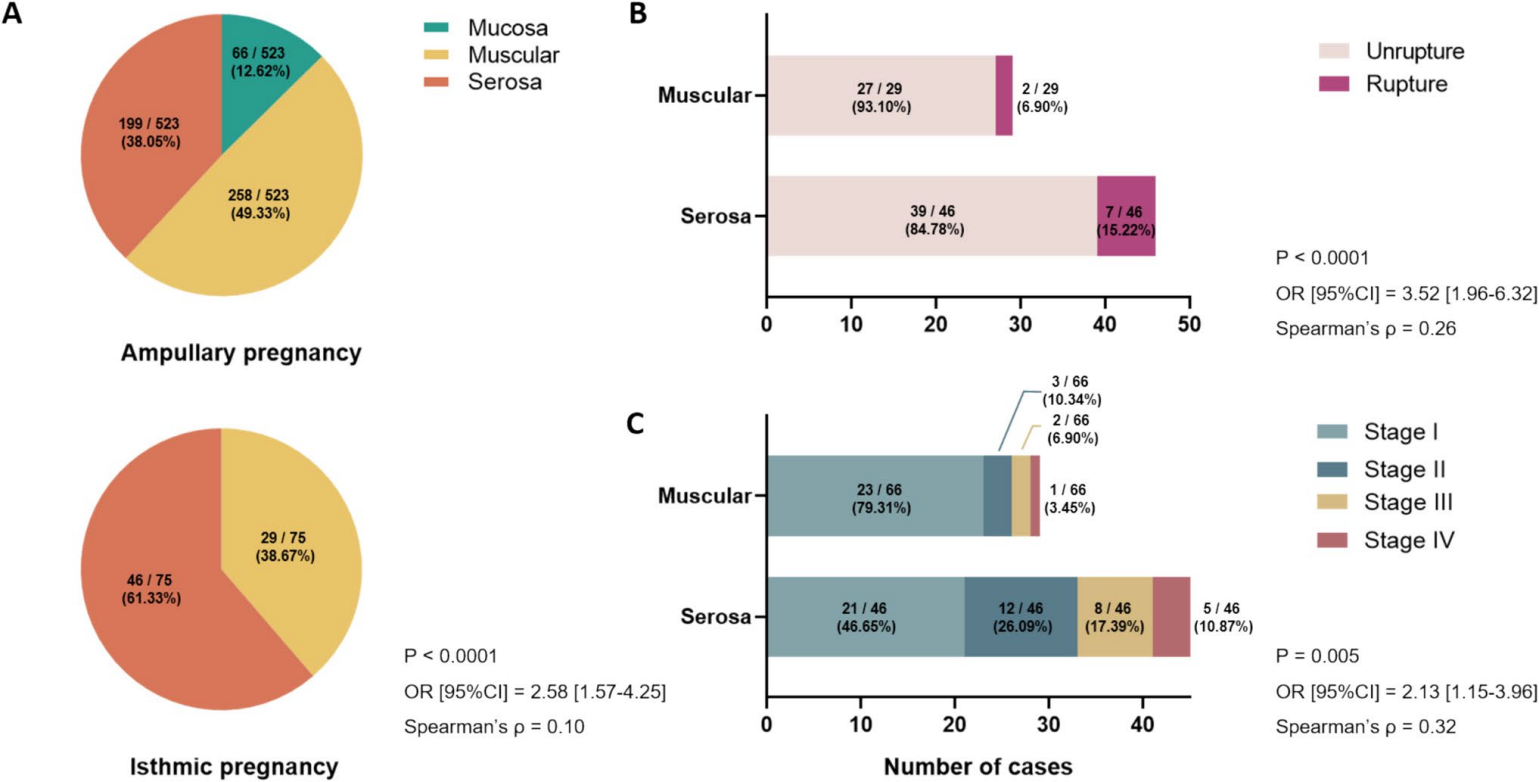
Correlation analysis of related characteristic of isthmic pregnancy. **A** Association of serosa infilatration with anatomic site of ectopic pregnancy (P < 0.0001, OR [95%CI] = 2.58 [1.57–4.25], Spearman’s ρ = 0.10). **B**. Association of tubal rupture with trophoblastic infiltration in isthmic pregnancy (P < 0.0001, OR [95%CI] = 3.52 [1.96–6.32], Spearman’s ρ = 0.26). **C** Association of trophoblastic infiltration with stages of vascular recast in isthmic pregnancy (P = 0.005, OR [95%CI] = 2.13 [1.15–3.96], Spearman’s ρ = 0.32)

Table 2 outlines the pathological patterns of fallopian tubes in isthmic and ampullary pregnancies, categorized into mucosa, muscular, and serosa groups based on trophoblastic infiltration depth. Notably, no cases of mucosal infiltration were observed in isthmic pregnancy in this study. Trophoblastic infiltration into the serosa was significantly associated with implantation site (OR [95%CI] = 2.58 [1.57–4.25], Spearman's *ρ* = 0.10, Fig. 5.A). Notably, in isthmic pregnancy, women with serosal infiltration exhibited deeper trophoblastic vascular remodeling compared to muscular infiltration (*P* = 0.04, Table 2). Additionally, although without significant difference, the incidence of SIN was lower in serosal infiltration compared to muscular infiltration in isthmic pregnancy(21.74% vs. 41.38%, *P* = 0.12). Furthermore, correlation analysis revealed a significant relationship between tubal rupture in isthmic pregnancy and trophoblast invasion depth (OR [95%CI] = 3.52 [1.96–6.32], Spearman's *ρ* = 0.26, Fig. 5.B) as well as the depth of vascular remodeling (*P* < 0.01, Spearman's *ρ* = 0.32, Table 3). It is also worth noting that as the infiltration of trophoblasts deepens, the stage of vascular remodeling increases in isthmic pregnancy, which also had a significant relationship (OR [95%CI] = 2.13 [1.15–3.96], Spearman's *ρ* = 0.32, Fig. 5.C).

**Table 2:** Comparison of pathological patterns of women between isthmic and ampullary pregnancy

No. (%)	Isthmic (*n* = 75)			Ampulla (*n* = 523)				P_3_ value
	Muscularis (n = 29)	Serosa (n = 46)	P_1_ value	Mucosa (n = 66)	Muscularis (n = 258)	Serosa (n = 199)	P_2_ value	
**Abnormalities of fallopian tube nearby the implantation site**								
**Chronic inflammation of the tube**								
No	24 (82.76)	42 (93.33)	0.30	40 (60.61)	132 (51.56)	136 (71.58)	< 0.01	< 0.01
Yes	5 (17.24)	3 (6.67)		26 (39.39)	124 (48.44)	54 (28.42)		
**Salpingitis isthmica nodosa**								
No	17 (60.71)	36 (78.26)	0.12	50 (75.76)	218 (84.50)	189 (94.97)	0.18	0.02
Yes	12 (41.38)	10 (21.74)		16 (24.24)	40 (15.50)	10 (5.03)		
**Edema villi**								
No	22 (75.86)	39 (84.78)	0.37	65 (98.48)	215 (83.33)	154 (78.17)	< 0.01	0.74
Yes	7 (24.14)	7 (15.22)		1 (1.52)	43 (16.67)	43 (21.83)		
**Degeneration of trophoblasts**								
No	26 (89.66)	39 (84.78)	0.80	56 (84.85)	209 (81.01)	179 (89.95)	0.03	0.73
Yes	3 (10.34)	7 (15.22)		10 (15.15)	49 (18.99)	20 (10.05)		
**Nucleated fetal red blood cells in the villi vessels**								
No	18 (62.07)	31 (67.39)	0.80	60 (90.91)	229 (88.76)	144 (73.10)	< 0.01	< 0.01
Yes	11 (37.93)	15 (32.61)		6 (9.09)	29 (11.24)	53 (26.90)		
**Stages of vascular recast**								
I	23 (79.31)	21 (46.65)	0.04	64 (95.45)	241 (93.41)	91 (45.73)	< 0.01	< 0.01^a^
II	3 (10.34)	12 (26.09)		2 (3.03)	5 (1.94)	42 (21.11)		
III	2 (6.90)	8 (17.39)		0 (0.00)	12 (4.65)	42 (21.11)		
IV	1 (3.45)	5 (10.87)		0 (0.00)	0 (0.00)	24 (12.06)		

P_1,2_ value is compared among different infiltration depth in isthmic and ampullary pregnancy, respectively; P_3_ value is compared between isthmic and ampullary pregnancy

^a^Tested by Mann–Whitney U Test

**Table 3:** Association of tubal rupture with stages of vascular recast in isthmic pregnancy^a^

No. (%)	Rupture	Unrupture
**Grages of vascular recast**		
I (n = 44)	1 (2.27)	43 (97.73)
II (n = 15)	5 (33.33)	10 (66.67)
III (n = 10)	1 (10.00)	9 (90.00)
IV (n = 6)	2 (33.33)	4 (66.67)

^a^ *P* < 0.01, Spearman’s *ρ* = 0.32

### Incidence of Chronic Inflammation

The incidence of chronic inflammation was lower when trophoblastic infiltration was deeper in ampullary pregnancy. Interestingly, the incidence of chronic inflammation in the fallopian tube was significantly lower in isthmic pregnancy compared to ampullary pregnancy (10.67% vs. 39.01%, *P* < 0.01, Table 2).

### Multivariate Analysis of Trophoblastic Implantation Site

Multivariate analysis (Table 4), adjusting for significant confounding factors identified in univariate analysis (Table 1 and Table 2, including clinical symptoms, adnexal mass size, ectopic germ on ultrasound, intraoperative ipsilateral tubal adhesions, pathological sections showing nucleated fetal red blood cells, and vascular remodeling), revealed that isthmic pregnancy was associated with a longer time from LMP to surgery (AOR = 1.07, 95% CI 1.03–1.12, *P* < 0.01) and higher serum β-hCG levels (AOR = 2.77, 95% CI 1.24–6.18, *P* = 0.01) when compared to ampullary pregnancy. Additionally, the occurrence of isthmic pregnancy was positively correlated with salpingitis isthmica nodosa (AOR = 3.62, 95% CI 1.45–9.00, *P* < 0.01) and negatively correlated with the presence of chronic inflammation of the fallopian tube (AOR = 0.24, 95% CI 0.09–0.64, *P* < 0.01) compared to ampullary pregnancy.

**Table 4:** Multivariate regression analysis of tubal isthmic pregnancy

	B	Standard error	Wald	Adjusted OR [95% CI]	*P* value
**Chronic inflammation of the tube**					
No	Reference				
Yes	−1.42	0.49	8.23	0.24 [0.09—0.64]	< 0.01
**Salpingitis isthmica nodosa**					
No	Reference				
Yes	1.29	0.47	7.65	3.62 [1.45—9.00]	< 0.01
**Time from LMP to surgery**	0.07	0.02	11.47	1.07 [1.03—1.12]	< 0.01
**Last β-hCG before surgery (IU/L)**					
< 3000	Reference				
≥ 3000	1.02	0.41	6.15	2.77 [1.24—6.18]	0.01

*OR* odds ratio

## Discussion

### Clinical Significance of Isthmic Pregnancy

Isthmic pregnancy has long been perceived as a more concealed and perilous form of ectopic pregnancy, with a higher propensity for rupture compared to ampullary pregnancy. This clinicopathological study sheds light on several critical aspects of isthmic pregnancy. Notably, women with isthmic pregnancy exhibited superior trophoblastic activity, deeper vascular remodeling, and more accessible trophoblastic infiltration into the deep tubal wall. Intriguingly, chronic inflammation of the fallopian tubes may not be a significant risk factor for isthmic pregnancy, while the presence of salpingitis isthmica nodosa (SIN) appears to be associated with its occurrence. To our knowledge, this study represents the first report on the interplay between chronic fallopian tube inflammation and ectopic pregnancy implantation site. Furthermore, it is among the few clinicopathological investigations conducted on a sizable sample of isthmic pregnancies in recent years.

### Comparison with Existing Literature

#### Salpingitis Isthmica Nodular and Isthmic Pregnancy

Notably, our observations indicate that isthmic pregnancies in our study did not show infiltration limited to the mucosal layer, and the last preoperative serum β-hCG levels were significantly higher for isthmic pregnancies compared to ampullary pregnancies. This suggests that isthmic pregnancies are more elusive and often diagnosed at a later stage when trophoblast infiltration is more advanced. The anatomical features of the isthmic, such as its narrow lumen and thin mucosal folds, likely contribute to this pattern. These factors, in conjunction with the deeper infiltration observed, suggest a higher propensity for rupture and a subsequent increased risk to women's lives. Moreover, it raises the possibility that distinct risk factors may underlie isthmic and ampullary pregnancies. Isthmic pregnancy may not be closely linked to chronic tubal inflammation, suggesting the involvement of alternative pathogenic factors. Our findings corroborate previous reports indicating a close relationship between SIN and isthmic pregnancy [20–23]. Histologically, isthmic nodules consist of hypertrophic tubal muscle layers and tubal epithelial glands, characterized by muscular hypertrophy. SIN primarily represents a non-inflammatory abnormality in the tubal wall structure, reinforcing the notion that isthmic pregnancy may be more influenced by tubal wall anatomical abnormalities. Interestingly, although not statistically significant, our study suggested that SIN predominantly occurs in the muscular layer of the fallopian tube wall, where muscular hypertrophy and a tortuous lumen may limit trophoblast cell infiltration into the serosal layer.

#### Association with Chronic Inflammation of the Fallopian Tube

Chronic inflammation of the fallopian tube is widely acknowledged as a primary risk factor for tubal pregnancy, with extensive research dedicated to elucidating the mechanisms through which inflammatory factors contribute to this condition [16–19]. Intriguingly, our prior study [4] speculated that fallopian tubes exhibiting chronic inflammation may limit trophoblast development and infiltration, ultimately resulting in the abortion of approximately two-thirds of tubal pregnancies. Conversely, in relatively normal fallopian tubes, well-developed trophoblasts infiltrate deeply, culminating in tubal rupture. However, scant attention has been directed toward exploring the relationship between chronic tube inflammation and the specific implantation site of tubal pregnancies. As far back as 1988, M. Senterman et al. conducted an early pathological study on tubal pregnancy [6], noting that trophoblasts penetrated the tubal wall earlier in isthmic pregnancy than ampullary pregnancy. Their study reported chronic inflammation in 28.57% of isthmic pregnancy cases compared to 56.82% in ampullary pregnancy. However, their study had a limited sample size (only seven cases of isthmic pregnancy) and did not incorporate clinical information for comparison [6]. In contrast, the current study, focusing on clinicopathological characteristics, with a larger sample size, revealed chronic inflammation incidences of 8/74 (10.81%) in isthmic and 204/512 (39.84%) in ampullary pregnancies, aligning with our previous findings (39.76%) in ampullary pregnancies [4], and underscores that the relationship between isthmic pregnancy and the fallopian tube's anatomical structure may be more substantial than its association with chronic inflammation.

#### Pathological Patterns and Association with Rupture Risk

The pathological patterns in ampullary pregnancies were consistent with our previous study [4]. Our study also revealed a higher probability of serosal infiltration in isthmic pregnancy compared to ampullary pregnancy. Additionally, as trophoblastic infiltration depth increased, vascular remodeling became more profound. Our previous research has shown that deep trophoblastic infiltration is linked to tubal pregnancy rupture [4]. The well-established risk of tubal isthmic pregnancy rupture compared to ampullary pregnancy aligns with previous literature [2, 6–8]. The narrow and non-expandable nature of the fallopian tube isthmus has been proposed as a contributing factor to this increased risk. Moreover, higher serum β-hCG levels have been associated with an elevated likelihood of isthmic pregnancy rupture. Collectively, our findings suggest potential interconnections among the depth of vascular remodeling, trophoblastic infiltration, and tubal pregnancy rupture, although no statistically significant differences in tube ruptures or active bleeding were observed between the groups (Table 1: 12.00% versus 9.18%, *P* = 0.53; 8.00% versus 4.97%, *P* = 0.42). Consequently, isthmic pregnancy, despite its lower incidence compared to ampullary pregnancy, warrants careful clinical observation and timely intervention based on individual cases. Future research should focus on exploring the pathogenic factors and pathological mechanisms specific to isthmic pregnancy to elucidate its unique characteristics and potential risks.

## Strengths and Limitations

This study stands out as the pioneer in investigating factors associated with trophoblastic infiltration in isthmic pregnancy from a clinicopathological perspective in recent years. No prior research has delved into the relationship between implantation sites of tubal pregnancy and chronic fallopian tube inflammation through histological examination. Furthermore, the substantial sample size of isthmic pregnancies included in this study surpasses that of comparable pathological investigations currently available. However, we acknowledge that the overall sample size remains relatively small, which may limit the generalizability of our findings. While the findings of this study are promising and contribute to our understanding of ectopic pregnancy mechanisms, several limitations must be acknowledged. This retrospective study relied on clinical data extracted from a database, introducing the potential for information bias. Additionally, due to constraints related to pathological materials, it was not feasible to ensure precise determination of implantation sites for each specimen, further potentially impacting the results. Moreover, as a single-center study with specific inclusion criteria, the pathological findings may not fully represent the broader population of tubal pregnancies, highlighting the need for multicenter and prospective studies in the future.

## Clinical Implications

Compared to ampullary pregnancy, isthmic pregnancy exhibits a delayed onset of symptoms, heightened trophoblastic activity, deeper vascular remodeling, more accessible trophoblastic infiltration into the tubal wall's deep layers, and an elevated risk of tubal rupture. In clinical practice, early identification and intervention are imperative to prevent life-threatening hemorrhage resulting from tubal rupture. Traditionally, isthmic pregnancy with high β-hCG have often led to surgical removal of the fallopian tube due to the narrow lumen and a high rupture risk. However, our study reveals that chronic fallopian tube inflammation is infrequent in isthmic pregnancy. Therefore, the structure and function of the fallopian tube adjacent to the implantation site may remain intact. Given the high incidence of SIN and the isthmic's abnormal structure, it is worth exploring whether a more conservative surgical approach, such as tubal segmental resection, could be suitable for villi removal while preserving the fallopian tube's reproductive function. However, this hypothesis is not directly supported by the findings of this study. Further investigations are needed to evaluate the feasibility, safety, and efficacy of such approaches before they can be recommended for clinical practice.

We contend that despite the existing evidence pointing to chronic fallopian tube inflammation as a significant risk factor for tubal pregnancy, the mechanisms governing isthmic pregnancy and ampullary pregnancy may differ. The relationship between isthmic pregnancy and chronic fallopian tube inflammation may not be as straightforward as previously thought. Future research should focus on conducting additional clinical, pathological, and basic studies to delve deeper into the underlying mechanisms of isthmic pregnancy.

In clinical practice, while early screening and timely intervention for tubal ectopic pregnancies are crucial, distinguishing between isthmic and ampullary pregnancies remains challenging with commonly used diagnostic tools such as ultrasound. The inherent limitations of these imaging techniques may hinder the implementation of heightened clinical vigilance as recommended. Hysterosalpingography (HSG), however, has been recognized as a valuable assessment tool, particularly for evaluating the condition of the contralateral fallopian tube following an ectopic pregnancy. Previous studies have highlighted the importance of post-operative HSG in assessing tubal pathology and identifying structural abnormalities that may impact future fertility. Incorporating HSG into clinical practice could provide critical insights into tubal health, guide reproductive counseling, and facilitate comprehensive decision-making for the management of ectopic pregnancies. Although our study did not specifically investigate the role of HSG in distinguishing isthmic from ampullary pregnancies, we acknowledge its potential utility in this context. Future research should explore the role of HSG both as a diagnostic tool and as part of post-operative assessment protocols to optimize clinical outcomes and fertility management following tubal ectopic pregnancies.

## Conclusion

Chronic inflammation of the fallopian tubes may not emerge as a risk factor for isthmic pregnancy, while the presence of salpingitis isthmica nodosa (SIN) appears to be associated with its occurrence. In comparison to ampullary pregnancy, isthmic pregnancy demonstrates a more profound degree of trophoblastic vascular remodeling in the fallopian tube, coupled with a heightened propensity to infiltrate into the tubal wall's deep layers. These factors render it a more concealed and perilous form of ectopic pregnancy, necessitating early recognition and diagnosis in clinical practice.

## Data Availability

The data that support the findings of this study are available from the corresponding author upon reasonable request.
